# Risk factors associated with sustained circulation of six zoonotic arboviruses: a systematic review for selection of surveillance sites in non-endemic areas

**DOI:** 10.1186/s13071-019-3515-7

**Published:** 2019-05-27

**Authors:** Helen J. Esser, Ramona Mögling, Natalie B. Cleton, Henk van der Jeugd, Hein Sprong, Arjan Stroo, Marion P. G. Koopmans, Willem F. de Boer, Chantal B. E. M. Reusken

**Affiliations:** 1 Resource Ecology Group, Wageningen University & Research, Wageningen, The Netherlands; 2 Present Address: Laboratory of Entomology, Wageningen University & Research, Wageningen, The Netherlands; 3 Department of Viroscience, WHO CC for arbovirus and viral hemorrhagic fever reference and research, Erasmus University Medical Centre, Rotterdam, The Netherlands; 4 Centre for Infectious Disease Control, National Institute for Public Health and Environment (RIVM), Bilthoven, The Netherlands; 5 Vogeltrekstation-Dutch Centre for Avian Migration and Demography, Netherlands Institute of Ecology (NIOO-KNAW), Wageningen, The Netherlands; 6 Centre for Monitoring of Vectors (CMV), National Reference Centre (NRC), Netherlands Food and Consumer Product Safety Authority (NVWA), Ministry of Economic Affairs, Wageningen, The Netherlands

**Keywords:** West Nile virus, Japanese encephalitis virus, Rift Valley fever virus, Tick-borne encephalitis virus, Louping-ill virus, Crimean-Congo hemorrhagic fever virus

## Abstract

Arboviruses represent a significant burden to public health and local economies due to their ability to cause unpredictable and widespread epidemics. To maximize early detection of arbovirus emergence in non-endemic areas, surveillance efforts should target areas where circulation is most likely. However, identifying such hotspots of potential emergence is a major challenge. The ecological conditions leading to arbovirus outbreaks are shaped by complex interactions between the virus, its vertebrate hosts, arthropod vector, and abiotic environment that are often poorly understood. Here, we systematically review the ecological risk factors associated with the circulation of six arboviruses that are of considerable concern to northwestern Europe. These include three mosquito-borne viruses (Japanese encephalitis virus, West Nile virus, Rift Valley fever virus) and three tick-borne viruses (Crimean-Congo hemorrhagic fever virus, tick-borne encephalitis virus, and louping-ill virus). We consider both intrinsic (e.g. vector and reservoir host competence) and extrinsic (e.g. temperature, precipitation, host densities, land use) risk factors, identify current knowledge gaps, and discuss future directions. Our systematic review provides baseline information for the identification of regions and habitats that have suitable ecological conditions for endemic circulation, and therefore may be used to target early warning surveillance programs aimed at detecting multi-virus and/or arbovirus emergence.

**Electronic supplementary material:**

The online version of this article (10.1186/s13071-019-3515-7) contains supplementary material, which is available to authorized users.

## Background

The recent (re-)emergence of different arthropod-borne viruses in Europe has highlighted the fact that both novel and known arboviral diseases can infest and establish in previously naive areas [1]. Globalization, climate change, habitat modification, and increased human and animal populations and movement (e.g. host dispersal, migration, transport) are only some of the factors that have redefined the geographical distribution of arboviruses and their vectors, such as ticks and mosquitoes [2]. A recent example is the dengue epidemic in Madeira in 2012, where the establishment of the (sub)tropical mosquito vector *Aedes aegypti* facilitated a dengue outbreak upon introduction of the virus by a traveler returning from an endemic country [3]. Multiple examples of introduction and/or geographical expansion of arboviral diseases and their vectors in Europe underline the need for effective surveillance in high-risk areas to maximize early detection, a prerequisite for adequate risk management [4–10].

Identifying areas with endemic potential, and hence improving the selection of areas for surveillance, requires a thorough understanding of the ecological factors (both biotic and abiotic) that promote local virus circulation. Due to the nature of their transmission cycle, which involves specific arthropod vectors and vertebrate hosts, the epidemiology of arboviruses is complex and strongly influenced by environmental conditions and other aspects of vector and host ecology. Arboviruses like yellow fever virus, dengue virus and chikungunya virus even circulate in different ecological cycles (i.e. sylvatic and urban cycles) involving different vector and host species and driven by differences in ecological factors such as vector host preferences, local host abundances and herd immunity [11–14]. Vector survival and arbovirus replication are strongly influenced by climatic conditions such as temperature, humidity and precipitation, with vector activity and virus transmission often being seasonal [15]. Local vector abundance may further depend on the availability of specific breeding habitats, such as water bodies for mosquito oviposition [16], or specific vertebrate host species for feeding [17]. For example, many tick species have different host preferences at different life stages [18], and high host densities may greatly increase tick abundance [19]. Further, host migration enables dispersal of both ticks and arboviruses over long distances, often to novel areas [17, 20]. Virus circulation is also influenced by the spatial and temporal variation in the densities of competent vertebrate reservoir hosts. Finally, the availability of suitable habitat and/or important host species is often correlated with particular land use categories, such as fragmented and highly mosaic agricultural landscapes [21]. However, despite recent advancements, our current understanding of the specific ecological factors that facilitate virus circulation and their relative impact thereon is limited for many arboviruses, and identification of areas of potential emergence therefore remains a challenge.

Here, we systematically review the ecological risk factors associated with the circulation of six arboviruses that are of considerable concern to northwestern Europe. Selection of these arboviruses was based on an earlier assessment of the risk of emerging zoonotic pathogens for public health in the Netherlands [22], and include the three top ranking mosquito-borne viruses, i.e. West Nile virus (WNV), Japanese encephalitis virus (JEV), Rift Valley fever virus (RVFV), and the three top-ranking tick-borne viruses, i.e. tick-borne encephalitis virus (TBEV), louping-ill virus (LIV) and Crimean-Congo hemorrhagic fever virus (CCHFV). While some of these viruses are already endemic to parts of Europe (WNV, TBEV, LIV and CCHFV), their ranges have recently expanded and may continue to spread both northwards and to higher altitudes under projected changes in climate, land use, and vector and host distributions [8, 17, 23, 24]. In contrast, JEV and RVFV are not yet circulating in Europe, but the potential for their introduction [25, 26] and/or the presence of putatively competent vectors [27, 28] in Europe merits further investigation. The results of our systematic review can be used for the identification of areas with the highest potential for virus introduction and establishment of circulation, thereby providing a rationale for targeted surveillance programs aimed at early detection of these arboviruses.

## Methods

### Data sources and search strategy

We performed a systematic literature search following the Preferred Reporting Items for Systematic Reviews and Meta-Analyses (PRISMA) guidelines [29] and Cochrane Collaboration guidelines [30]. We searched for peer-reviewed articles in PubMed and EMBASE databases (Table 1). We first screened potentially relevant records based on titles, abstracts and keywords and then read full-text articles to evaluate them according to our selection criteria.

**Table 1:** Search terms

Search term	Alternatives
West Nile virus	“West Nile virus”[Text] OR “West Nile fever”[Text] OR “WNV”[Text]
Japanese encephalitis virus	“Japanese encephalitis virus”[Text] OR “JEV”[Text] OR “Japanese encephalitis”[Text] OR “Japanese B encephalitis”[Text]
Tick-borne encephalitis virus	“Tick-borne encephalitis virus”[Text] OR “FSME”[Text] OR “FSME-virus”[Text] OR “Frühsommer-Meningoenzephalitis”[Text] OR “TBEV”[Text] OR “Tick-borne encephalitis”[Text] OR “TBEV-Eu”[Text] OR “TBEV-Sib”[Text] OR “TBEV-FE”[Text] OR “Louping ill virus”[Text] OR “LIV”[Text] OR “Ovine Encephalomyelitis”[Text]
Crimean Congo hemorrhagic fever	“CCHFV”[Text] OR “Crimean Congo”[Text] OR “Crimean hemorrhagic fever”[Text] OR “Crimean haemorrhagic fever”[Text] OR “Crimean Fever”[Text] OR “CCHF”[Text] OR “Hemorrhagic Fever Virus, Crimean-Congo”[Text] OR “Hemorrhagic Fever Crimean”[Text]
Rift Valley fever virus	“Rift valley fever virus”[Text] OR “RVFV”[Text] OR “Rift Valley”[Text] OR “RVF”[Text] OR “Rift Valley Fever”[Text]
Ecological study	“Transmission cycle”[Text] OR “ecology”[Text] OR “ecological”[Text] OR “risk factor”[Text] OR “risk model”[Text] OR “ecological study”[Text] OR “ecological competence”[Text] OR “natural ecology”[Text] OR “enzootic cycle”[Text] OR “epizootic cycle”[Text] OR “urban cycle”[Text] OR “Risk-modifying factor” [Text] OR “dynamics”[Text] OR “competence” [Text] OR “Cycle” [Text] OR “environmental factor”[Text]
Habitat	“Forest”[Text] OR “wetlands”[Text] OR “shrubs”[Text] OR “trees”[Text] OR “bushes”[Text] OR “environment”[Text] OR “urban”[Text] OR “river”[Text] OR “marshes”[Text] OR “grass”[Text] OR “grassland”[Text] OR “water reservoirs”[Text] OR “natural ecology”[Text] OR “habitat”[Text] OR “green”[Text] OR “landscape”[Text], OR “habitat”[Text], OR “vegetation type”[Text]
Climate	“Temporal”[Text] OR “temperature”[Text] OR “humidity”[Text] OR “rain”[Text] OR “precipitation”[Text] OR “water”[Text] OR “weather”[Text] OR “climate” [Text]
Geography	“Spatial”[Text] OR “density”[Text] OR “concentration”[Text] OR “movement”[Text] OR “migration”[Text] OR “abundance”[Text] OR “population and size”[Text]

### Selection criteria

Studies were considered eligible if they met the following selection criteria: (i) studies focused on ecological variables that may promote virus circulation, including variables related to climate (e.g. precipitation, humidity, temperature), habitat (e.g. habitat fragmentation, Normalized Difference Vegetation Index), or vector and host ecology (e.g. population density, migration); (ii) studies reported only primary collected data or models; (iii) studies published between January 1st 1980 and January 7th 2016.

Reviews were excluded but all the data they reported were checked and compared to the results of our literature search. Any study that was identified in the reviews but not selected by our search was added to our review. Bibliographies of relevant articles and literature reviews were also cross-checked for potential relevant records. We used a data extraction pre-piloted form and entered the extracted data into an Excel database. Data published in multiple articles were included only once. Data from articles published in English were extracted from the full-texts, while data from records published in other languages were extracted from the English abstract or excluded when no English abstract was available.

## Results

**Fig. 1:**
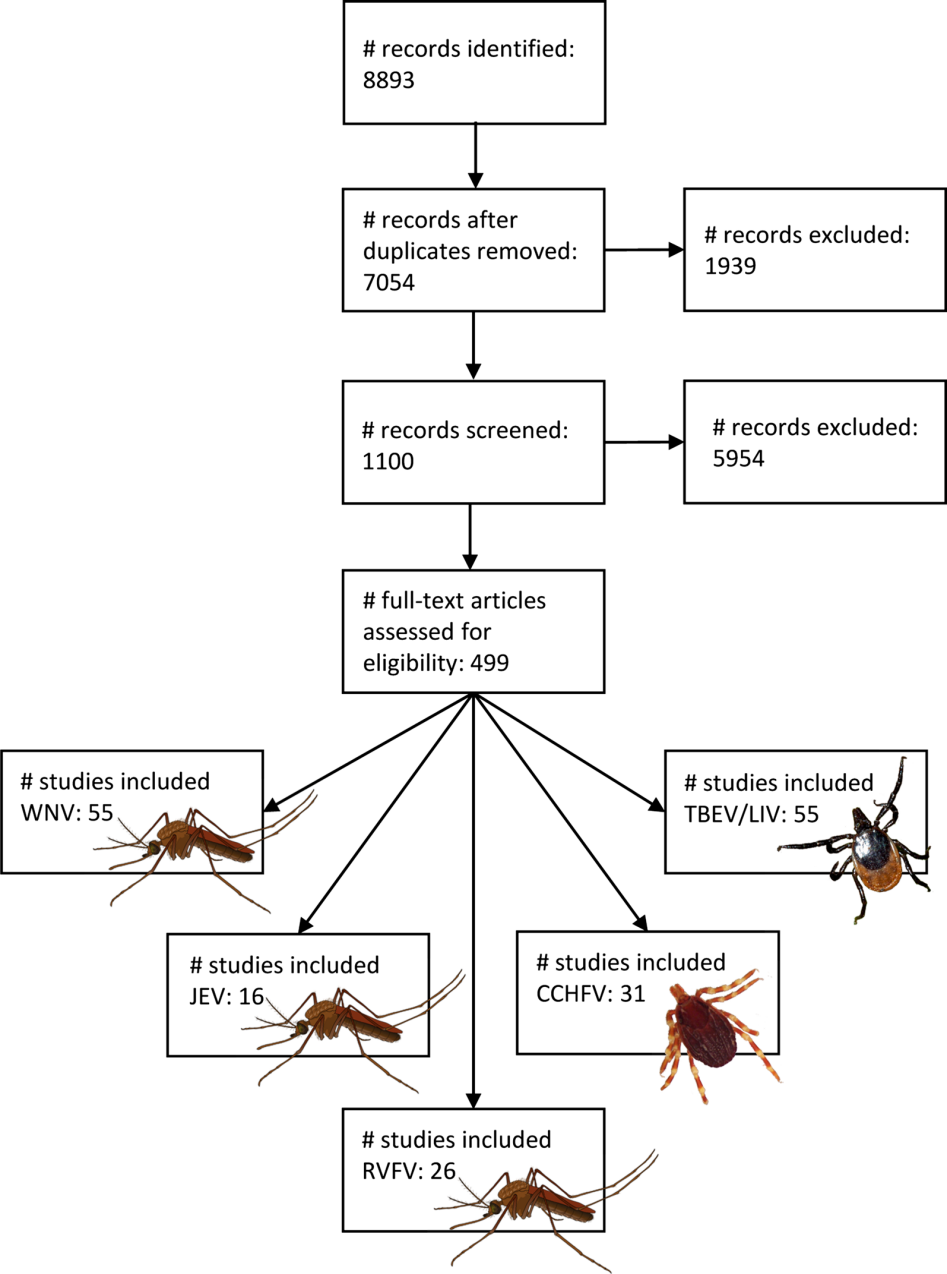
PRISMA flow diagram of the literature search

The PRISMA flow diagram of our literature search to review ecological risk factors associated with the circulation of the six priority viruses in northwestern Europe is given in Fig 1. Background information on each virus can be found in Additional file 1.

### CCHFV

Climatic factors were often invoked to explain the upsurge of CCHFV cases in recent years, as they strongly influence the survival, development, and reproduction of the principal vector, *H. marginatum* (*s.l*.) [31, 32]. A dynamic population model of *H. marginatum* showed that ticks were unable to establish self-sustaining populations in areas where the yearly sum of the average daily temperature falls below 3000–4000 °C, a limit roughly found at latitudes north of 47°N [31]. Below this temperature limit, most engorged nymphs appear in late summer and early autumn. Since the molting process is only completed after 300 cumulative degrees Celsius above the developmental zero (14–16 °C, the minimum temperature needed for development), these nymphs are unable to molt into adults before winter and suffer higher mortalities as a result [33]. Indeed, cumulative temperatures between September and December average 800 °C in areas where *H. marginatum* has permanent populations, while the species is absent in areas where it drops below 400 °C [33]. Tick populations are also unable to persist at latitudes south of 34°N, as engorged nymphs in these areas tend to molt in late spring and early summer, when humidity levels are too low to maintain water balance [31]. Thus, annual cumulative temperature appears to be an important barrier to further spread of *H. marginatum*, and hence CCHFV distribution [34]. However, estimates of future climate change scenarios may sharply increase suitable habitat and hence facilitate its northwards spread [35].

*Hyalomma marginatum* ticks become active in spring when average daily temperatures exceed 10.5 °C, with optimal development between 22–27 °C and 75–100% relative humidity under laboratory conditions [36]. Tick activity lasts until early autumn [37]. In Turkey, increased tick activity in summer coincided with CCHF incidence in humans, with most cases concentrated from May through July, when mean monthly temperatures ranged between 15–20 °C [38]. In Iran, CCHF incidence peaked at mean monthly temperatures between 30–40 °C and with maximum relative humidity levels of 20–50% [39].

Various empirical and modelling studies have demonstrated that CCHFV risk areas are associated with a range of climatic factors permissive for establishment of *H. marginatum* populations [21, 32, 38–42], although others did not [43]. CCHF incidence in humans was positively related to mean monthly temperature in Turkey, Iran and Bulgaria [38, 39, 41, 42] and maximum relative humidity (RH) in Iran [39]. These correlations are likely to arise through the indirect impact of climate on tick life-cycle dynamics and survival. Higher temperatures and humidity facilitate higher tick development rates, transstadial virus transmission, increased tick questing activity, and higher host parasite loads, all of which promote virus circulation [32, 39].

Relationships between CCHFV and precipitation are more complex. Rainfall was positively associated with monthly CCHF incidence in Turkey, with mean monthly rainfall varying between 30–70 mm [38]. In contrast, in Senegal rainfall was negatively associated with CCHFV seropositivity in both humans and sheep, with IgG antibody prevalence dropping from 31% to 0% when annual precipitation increased from 450 mm to over 1000 mm [44]. In Iran, monthly accumulated rainfall (maximally 150 mm for each of the four seasons) was inversely correlated with CCHF incidence at a lag of one month, but positively correlated at a lag of five months [39]. These studies show that the relationship between CCHFV circulation and precipitation remains contentious and may depend on the climate zone, which in the given examples varied from Mediterranean, to tropical and arid regions, respectively.

Elevation was also related to CCHFV risk, with increased seropositivity in people living at altitudes of > 400 m [45] or > 600 m [46]. Dogan et al. [47] also found a strong, positive relationship between CCHF incidence and elevation, but only until an elevation of 1340 m, suggesting an altitude threshold for *Hyalomma* spp. ticks. Aker et al. [38] found the highest incidence of CCHF in humans at elevations between 1000–1200 m. However, as altitude is correlated with factors such as temperature, precipitation, vegetation, and host densities, the effect of altitude cannot be separated from the aforementioned factors so that the underlying causal mechanisms remain unclear.

Besides climatic conditions, a range of other environmental factors may contribute to higher levels of CCHFV circulation by increasing tick habitat suitability or seasonal activity. These factors are often related to particular land cover categories. For example, human cases of CCHF were positively related to the proportion of areas covered by shrub, grassland, and herbaceous vegetation [42, 48], the Normalized Difference Vegetation Index (NDVI) [42, 49], and high rates of habitat fragmentation in agricultural areas [21, 42, 43]. This corresponds to the preferred habitat of *Hyalomma* ticks: savanna and lightly wooded foothill biotopes [42, 50]. Intermingling of fragmented farmland with forested areas not only provides suitable habitat for ticks but may also stimulate movement of larger animals that carry adult ticks to new locations [21, 42, 51].

Increased densities of wild and domestic ungulates and (illegal) livestock trading were also found to be key factors for CCHFV spread throughout the western Palaearctic region [32, 38, 39], although livestock density was not always correlated with CCHF incidence in humans [42]. Human population density has not been shown to correlate with CCHF incidence, but activities that increase human interaction with potentially infectious animals did, such as hunting practices and animal husbandry [21, 41, 42, 45]. In fact, the major human risk groups for CCHFV are people who come into close contact with livestock, such as farmers and abattoir employees [39]. Environmental and social changes that increase the risk of CCHFV in humans may have varying causes, including war conflicts (e.g. past outbreaks of CCHFV occurred in Crimea during WWII and in Kosovo in 1999), changes in animal husbandry practices, agricultural reforms, and abandonment of arable land [17, 43, 52].

Finally, several *Hyalomma* species, including *H. marginatum*, have a two-host life-cycle in which ticks remain on their host while molting from larva to nymph [53]. These ticks may therefore be attached to their first host for several weeks, in contrast to three-host ticks which drop off after each blood meal, usually within a few days [54]. The two-host life-cycle of *H. marginatum* in combination with transovarial transmission of CCHFV favors long-distance dispersal of both vector and virus *via* migratory birds or livestock trade [31, 39, 55]. Although no evidence exists that wild birds become viremic, immature ticks are frequently introduced into northern latitudes by passerine birds during spring migration [56–58]. Adult *H. marginatum* ticks have also been found on a horse in the Netherlands [59] and on the clothing of a person in Germany [60], and these ticks may have initially arrived as a nymph and molted to the adult stage after arrival in the country *via* migratory birds [61].

### TBEV

Several studies have demonstrated that a high prevalence of co-feeding between uninfected larvae and infected nymphs of *Ixodes ricinus* ticks on rodent hosts is the most critical factor in TBEV transmission [62–69]. Interstadial co-feeding only occurs when the seasonal activity of nymphs and larvae coincides, for which a specific combination of climatic and host factors is required [62, 63]. Specifically, warm summers allow for rapid development of eggs, but rapidly decreasing temperatures in autumn sends the emergent larvae into behavioral diapause during winter. Subsequent rapidly rising temperatures and humidity in spring causes larvae to become active at the same time as nymphs, allowing for efficient virus transmission *via* co-feeding [62–64, 69]. Experimental studies have indeed shown that the ratio of larvae to nymphs on rodent hosts increase with RH [70], and that RH also positively affects TBEV infection rate and transstadial transmission in ticks [71]. In Norway, TBEV prevalence in ticks was highest in sites with the highest mean RH and lowest mean saturation deficit (SD) [72]. In the Czech Republic, TBE incidence in humans was positively correlated with daily mean, maximum and minimum temperature as well as soil temperature at a depth of 5 cm, probably mediated by the direct effect of climatic factors on the abundance and/or questing behavior of *I. ricinus* [73]. Indeed, microclimatic factors play a crucial role in the life-cycle of *I. ricinus*, with tick abundance being positively correlated with temperature and RH but negatively with SD [69, 74–78]. Conversely, the negative impact of altitude on tick abundance and TBE incidence is probably confounded by climatic factors, as lower temperatures at higher altitudes deplete a tick’s fat and water reserves, shorten tick questing period and development rates, and delay embryogenesis [79].

Climate change has often been implicated in the rise of TBEV [80–83], but the subject remains controversial [84–87]. Lindgren & Gustafson [88] showed that in Sweden, incidence of TBE in humans increased under a combination of (i) two consecutive mild winters; (ii) daily minimum temperatures favoring spring development (8–10 °C) and extended autumn activity (5–8 °C) in the preceding year; and (iii) spring temperatures that allow tick activity (5–8 °C) in the incidence year. Thus, the increase in TBE incidence in humans in Sweden since the 1980s may be related to a combination of milder winters and early arrival of spring, which are favorable conditions for the tick vector as well as host and reservoir animals [88]. However, Randolph [89] argued that there was no warming during the 1984 TBEV outbreak in Sweden. While increased temperatures did precede the dramatic increase of TBE cases in central and eastern European countries in 1993 [90, 91], the spatial-temporal heterogeneity of the TBEV upsurge could not be explained by climate change alone [86, 92]. Instead, a combination of biotic, abiotic and human factors has been identified, many of which related to the fall of the Soviet Union and subsequent socio-economic transition [92, 93]. The most significant of these variables was human behavior, both recreational and subsistence, that caused higher exposure to infected ticks [86, 93]. For example, tick bites increased when dry weekends were followed by wet weeks, irrespective of local tick abundance, suggesting that TBEV risk could be better explained by humans harvesting mushrooms, berries and other forest products than by climate *per se* [89].

Apart from shifts in climatic variables and human behavior, both empirical and modelling studies have shown the important role of large wildlife, particularly deer, in TBEV epidemiology [66, 68, 94–99]. Although deer are not competent for TBEV transmission [62], they are among the most important host species for adult *I. ricinus* ticks [100]. As a consequence, deer are able to greatly amplify tick abundances, but at the same time divert tick bites from small rodents, which are competent hosts [95]. These two competing effects result in a hump-shaped relationship of deer density with the number of ticks feeding on rodents, and hence also with the basic reproduction number R_0_ (the average number of secondary infections produced by an infected individual in a fully susceptible host population [101]) for TBEV [96, 98]. Thus, deer initially have a positive (amplification) effect on the number of co-feeding ticks and TBEV persistence until a certain threshold density is reached, after which tick bites are ‘wasted’ as the ratio of blood meals on competent *versus* incompetent hosts decreases, triggering a decline (dilution) of TBEV prevalence in ticks [68, 98]. In addition, deer are able to transport infected ticks into previously naive populations [102, 103], as do (migratory) birds [104].

Well-connected broad-leaf and mixed forest patches with open areas were also positively correlated with the presence of TBEV, as they provide suitable habitat for roe deer, rodents and ticks [105, 106]. Although *I. ricinus* ticks are found in a wide range of habitats, they are most strongly associated with woodlands and forests with thick undergrowth, which offer favourable microclimatic conditions [107, 108]. Likewise, *Apodemus flavicollis*, the rodent most often incriminated in non-viremic TBEV transmission, is generally considered a species of mature forests with abundant undergrowth [97]. Changes in land management and forest structure that improve habitat suitability of forest rodents, as well as changes in hunting practices that affect roe deer densities are likely to play a major role in TBE upsurges, particularly in western Europe [97].

The contribution of each of these factors to local virus circulation may vary between regions. For example, a study from the Czech Republic argued that there were no socio-economic shifts, land-use changes, or changes in the abundance of game species that could explain the recent TBEV upsurge [82]. The authors suggested that human recreational activity (i.e. mushroom picking) and a warming climate were the most important drivers of the increase in TBE incidence [82, 109]. Further, a study from Sweden found a positive relationship between human cases of TBE and the number of red foxes *Vulpes vulpes* [110]. The authors suggested that red foxes may play a role in TBEV epidemiology by potentially acting (i) as reservoir hosts; (ii) as carriers and disseminators of ticks; (iii) as predators that directly suppress the abundance of incompetent hosts; and/or (iv) as mesopredators that indirectly facilitate competent hosts by suppressing the abundance of small carnivores, which prey upon small rodents [110]. Hence, the relative role of the most important factors—climate change, human behavior, relative abundance of competent *vs* incompetent hosts—in determining TBEV circulation varies spatially.

### LIV

Very few empirical studies have considered the environmental (both biotic and abiotic) risk factors associated with LIV circulation in endemic areas [79, 111, 112]. However, given that LIV is closely related to TBEV and is transmitted by the same tick vector (*I. ricinus*), many of the factors affecting TBEV circulation may also be involved in LIV transmission dynamics. For example, the distribution of LIV across the British Isles is tightly linked to those areas where its vector is most abundant, i.e. upland grazing areas [113]. Here, *I. ricinus* ticks become active when average daily spring temperature reaches 7 °C [113]. The abundance of larvae and nymphs on the British Isles was also found to be positively associated with deer densities [79]. In addition, nymph and adult tick abundances were strongly negatively associated with altitude after correcting for biotic factors such as hosts and vegetation, a relationship most likely explained by decreasing temperatures and humidity with increasing elevation [79]. Larval and nymphal *I. ricinus* both exhibit a similar seasonal pattern in abundance in parts of the LIV distribution, allowing for interstadial co-feeding transmission *via* mountain hares *Lepus timidus*, an essential element in LIV maintenance [62, 114].

Laurenson et al. [112] demonstrated the critical role of mountain hare in the LIV transmission cycle with a field experiment in the Scottish Highlands. Mountain hares were shown to be important amplifying hosts for ticks of all stages, and could maintain tick populations under low mountain hare densities and when large host species (e.g. deer, sheep) were either absent or treated with acaricides. Further, a reduction in mountain hare populations not only dramatically reduced tick loads but also LIV prevalence in red grouse *Lagopus lagopus scoticus* [112]. These results are in concordance with modelling studies of LIV transmission dynamics, which have shown that the virus could be maintained in the absence of red grouse through non-viremic transmission in mountain hares *via* co-feeding ticks [115, 116]. In contrast, the virus could not persist when red grouse density fell below 30 km^−2^ and mountain hares were absent [115]. Red grouse rarely feed adult ticks, so that LIV was only able to persist in conjunction with other species that function as reproduction hosts, such as red deer or mountain hares [112].

More recent modelling studies have shown the importance of incorporating spatial variation in host abundance and movement for predicting virus persistence and vector abundance [117]. Specifically, Watts et al. [117] showed that allowing a small proportion of the red deer *Cervus elaphus* population to move between sites with and without mountain hares allowed persistence of LIV at the hare-free site, even at very low deer densities. This may also explain why LIV prevalence in red grouse is not always reduced in sites where hare density is reduced [117]. In addition to movement of wildlife, movement of livestock has also been implicated in the spread of LIV. Phylogenetic analyses revealed that trade in cattle and sheep may account for the spread of LIV across the British Isles and its introduction into Norway [118].

### JEV

Temperature, humidity and precipitation stimulate the growth, survival and dispersal of JEV mosquito vectors as well as virus propagation [119, 120]. In China, incidence of JE in humans has a strong seasonal pattern, with cases rapidly increasing from June onwards, peaking in August and decreasing again from September onwards [120, 121]. Several empirical studies have shown this trend to be positively associated with monthly mean RH [122, 123], monthly mean temperature with lag times ranging from zero to three months [120–124], and yearly minimum and mean temperatures [125]. Epidemic peaks in China were also positively related to annual mean temperature (AMT), with only few cases when AMT was below 20 °C, potential epidemics when AMT was between 25–30 °C, and epidemic peaks when AMT was above 30 °C [120]. One study found lower threshold values of 25 °C and 21 °C for maximum and minimum temperature respectively, above which monthly JE incidence increased with temperature [122]. Temperature was also positively correlated with mosquito densities in JEV endemic areas in India, were optimal temperatures for JEV vectors ranged between 22.8–34.5 °C [126, 127]. Higher temperatures increase development and winter survival rates of mosquito larvae, while shortening the incubation period for virus replication within mosquitoes as well as time between blood meals. This reduces the transmission time from mosquito vector to animal and human hosts [128]. Thus, higher temperatures allow mosquito populations to increase more rapidly and to reach large, stable population sizes for longer, thereby facilitating JEV transmission [122, 129].

Relationships between JEV circulation and precipitation were more complex. While transmission of JEV occurs year-round in the tropics, seasonal epidemics typically start shortly after the onset of the rainy season when mosquito densities peak [129]. While several studies reported positive correlations between human cases and precipitation [120, 122, 124, 127], others found a negative relationship with lag times ranging from 0 to 4 months [121, 130], or a unimodal pattern with highest incidences at intermediate rainfall [125]. Chen et al. [131] found the effect of precipitation on JEV risk to be positive only up to a threshold value of 350 mm/day, above which mosquito breeding habitats were probably destroyed. However, these torrential rainstorms of > 350 mm/day are absent in temperate areas. This may also explain the negative relationship of rainfall with JE incidence in the study of Bai et al. [121] carried out in an area with a monsoon climate where annual precipitation exceeded 1000–1400 mm. An ecological niche modelling (ENM) study for *Cx. tritaeniorhynchus* in Asia, based on mosquito presence records and environmental variables, indicated that mean precipitation of the wettest quarters (797 mm) and elevation (153 m) were the most important contributors to the vector distribution model, with mosquitoes rarely occurring in areas above 1000 m [128]. An ENM study from China also reported a negative correlation between elevation and the presence of JE, possibly due to reduced temperatures and/or lower populations of humans, livestock, and ardeid birds [125].

Apart from climatic conditions, human incidence of JEV was positively related to pig density, the level of viremia in pigs, human proximity to pig farms, human population density, and the virulence of the virus [120, 124, 125, 128, 132, 133]. The principal mechanisms that allow JEV introduction and subsequent establishment in new areas include bird migration, movement and transportation of infected hosts (particularly pigs), the spread of mosquitoes by wind or trade (e.g. *via* ship deck cargo or aircraft), and changing land-use and agricultural practices following deforestation [129, 134–136]. Low lying, flooded areas such as rice paddies are the primary larval habitat for *Cx. tritaeniorhynchus* mosquitoes. The creation of new rice paddies or physical and chemical alterations to these breeding sites may have a strong influence on vector abundance and hence influence the establishment and/or spread of JEV [134]. In particular, paddy height, dissolved oxygen, and ammonia nitrogen have been shown to negatively impact larval abundances, whereas water temperature and nitrate nitrogen had consistent positive effects, both within and between seasons [16]. The expansion of rice growing areas over the past decades has increased adult mosquito population densities and may have subsequently contributed to human exposure to JEV [128].

### WNV

WNV ecology is controlled by a wide range of local mosquito, host reservoir and virus intrinsic factors, including vector competence, host preferences and longevity of mosquito vectors, morbidity, mortality and reservoir competence of vertebrate host species, and WNV strain virulence [55, 137–147]. The presence or absence of WNV in an area is determined by a complexity of interacting ecological factors, such as temperature, precipitation, and the simultaneous presence of sufficient densities of susceptible hosts and competent vectors [139, 140, 148, 149]. However, the direction and strength of correlations with these factors are not always consistent [149]. For example, laboratory studies have shown that WNV infection and transmission rates increase with temperature in some mosquito biotypes but not in others [139], and that some virus strains are transmitted more efficiently with increasing temperature than others [150]. Likewise, the effect of precipitation on WNV epidemiology differs between regions and may depend on vector species and lineage, as well as virus strain [149].

In the Danube Delta in Romania, warmer spring temperatures were positively correlated with mosquito densities and increased WNV infection rates in mosquitoes with a lag of 20 days from the onset of temperature rise. In contrast, precipitation was negatively correlated with mosquito infection rates with a lag of 30 days, whereas there was no correlation with mosquito densities [151]. While higher precipitation is generally believed to augment mosquito densities by increasing the availability of suitable breeding habitat, the opposite may be true for wetland ecosystems such as the Danube Delta [152]. Here, drought reduces water flow, thereby creating stagnant water pools with higher concentrations of organic matter, which are ideal conditions for mosquito breeding [149, 151, 153]. In addition, birds concentrating around small water holes during droughts can increase bird-mosquito interactions [149, 153]. A number of other studies have also found a link between increased WNV transmission and reduced precipitation [154–158] and increased temperatures during summer months [149]. In their study across the European continent, Marcantonio et al. [153] showed that WNV incidence was indeed positively correlated with high temperatures and drought during summer, but also with high precipitation in late winter/early spring.

In addition to climatic factors, variation in land use, host community, urbanization, and human behavior have been implicated as factors influencing WNV outbreaks [149, 153, 159–161]. For example, the percentage of irrigated crop lands and highly fragmented forests were positively related to WNV incidence across Europe [153]. Irrigated crop lands provide suitable breeding habitat for mosquito vectors, whereas forest fragmentation may increase contact rates between mosquitoes, reservoir hosts and humans [153]. A study from the Camargue wetlands, France, identified dry bushes, open water and woodlands as the major risk areas associated with WNV cases in horses, possibly by providing nesting and resting areas for birds [162]. Further, rice fields and reeds, which are ideal habitat for *Culex* spp. larvae, covered a large area within a 1 km buffer zone around localities from which WNV cases were reported [162]. In a follow-up study, Pradier et al. [163] showed a positive correlation between WNV circulation in southern France and one landscape metric (i.e. the interspersion and juxtaposition index, IJI) and one land cover class (i.e. heterogeneous agricultural areas). Both variables represent landscapes with spatial configurations that may facilitate interactions between competent vectors and reservoir hosts.

Many of the ecological processes that drive WNV outbreaks in the Old World differ from those of the New World [153], and seemingly inconsistent findings characterize both systems. As in Europe, relationships between WNV occurrence and specific intrinsic or extrinsic factors of vector, virus and host varied across the geographical range of WNV in the USA. For example, the correlation between human incidence of WNV and precipitation of the preceding year was found to be positive in the eastern USA, but negative in the west [157]. The authors hypothesized that differences in the ecology of mosquito vectors may be responsible for this difference, although rainfall patterns also differ between the east and west. Likewise, WNV incidence in the northeastern USA was positively associated with urban land cover [164], while in the western USA it was positively associated with agricultural land cover, a discrepancy that was explained by the geographical distribution of different WNV vectors [165]. Some *Culex* species tend to be well-adapted to anthropogenic environments, and urbanization may increase the number of water bodies that allow for mosquito oviposition, hence increasing vector abundances [159]. Indeed, Brown et al. [166] showed that within urban areas, the abundance of four important WNV vector species was positively correlated with proximity to water, NDVI, and Disease Water Stress Index (DWSI), which is a measure of the internal water content of vegetation. Further, warmer urban microclimates increased mosquito biting and development rates, increased the survival of overwintering virus-infected mosquitoes, and facilitated virus replication within mosquito vectors as well as transmission to susceptible hosts [23, 153, 160, 167, 168].

In contrast to Europe, several studies from the USA have suggested a potential role for avian diversity in reducing WNV transmission [159, 169–171], in line with predictions derived from the “dilution effect” hypothesis [172]. According to this hypothesis, high avian diversity reduces WNV transmission by redistributing vector blood meals across a much wider range of bird species, many of whom are poor reservoir hosts for WNV [169]. Negative correlations with avian diversity have been reported for WNV prevalence in mosquito vectors [169, 170], density of infected mosquitoes [170], and WNV incidence [169–171]. In contrast, Loss et al. [173] did not find support for a negative correlation of avian diversity with WNV seroprevalence in birds nor with mosquito infection rate. Moreover, the possibility of a dilution effect in WNV transmission has received considerable criticism, and these authors have argued that other intrinsic and extrinsic factors, such as heterogeneity in mosquito host preference, host reservoir competence, temperature, and precipitation, may be more important than avian species richness in driving local WNV transmission [173–175].

A striking difference between the USA and Europe, regarding the role of birds in WNV epidemiology, is the mass mortality of birds in the USA and lack thereof in Europe. Although WNV-neutralizing antibodies have been found in a wide range of wild bird species in Europe, their prevalence is usually rather low, and it remains unclear which species act as reservoir hosts [176–181]. Recent studies confirmed however that European carrion crows (*Corvus corone*) and Eurasian jackdaws (*Corvus monedula*) are as susceptible to WNV as American corvids [146, 182, 183]. This suggests that these birds, as well as closely related species such as the hooded crow (*Corvus cornix*) could also act as amplifying hosts in Europe [178, 183]. Other studies found high levels (11–70%) of antibodies in rock pigeons (*Columba livia*), suggesting that this species could facilitate the spread of WNV in Europe [23, 184]. Rock pigeons are abundant, fly long distances regardless of seasonal migrations, and, being an important prey of predatory birds, can contribute to oral transmission of WNV through the food chain [23]. A recent experimental study demonstrated that this species is indeed a competent reservoir host for WNV in Europe [185]. Figuerola et al. [178] found that body size of birds was a significant predictor for antibody prevalence. Larger hosts may be more readily infected with WNV due to their larger surface area and higher CO_2_ production, which may attract a larger number of mosquitoes.

While infected mosquitoes may spread WNV over long distances, either *via* unassisted means (e.g. by wind currents) or assisted means (e.g. *via* boats or aircraft) [186], the principal route for WNV dissemination is *via* infected migratory birds, which carry WNV to novel areas as far north as Sweden [179, 187]. In southern Spain, trans-Saharan migratory birds had both higher antibody prevalence and titers than short-distance migrants and resident bird species [180]. Although this does not provide definite proof that these specific bird species are involved in WNV-transmission, it does suggest that long-distance migrants spend part of their lives in African regions with greater circulation of WNV [6, 147, 176, 177, 188, 189]. Once introduced in favorable habitat, WNV may overwinter in infected female mosquitoes as well as in resident birds, removing the need for continuous re-introductions by migratory species [176, 190].

### RVFV

Historical outbreaks of RVFV since the 1950s in East Africa were closely linked to climate anomalies [191–196]. Epidemics and epizootics typically occur after periods of heavy rainfall and flooding, which create temporary mosquito breeding pools (“dambos”) that allow for the development of large numbers of mosquitoes [193, 197–201]. Periods of exceptional rainfall in East Africa are associated with the warm phase of the El Niño/Southern Oscillation phenomenon (ENSO) [202]. Analysis of past outbreaks have shown that over three quarters of RVFV events have occurred during warm ENSO periods [194, 203]. Another indicator of increased precipitation is greener vegetation, which can be detected by the satellite derived NDVI. Spatiotemporal analysis of NDVI, rainfall, and several indicators of ENSO have all been successfully used to predict RVFV outbreaks [194, 196, 204–206]. Yet some past outbreaks in other parts of Africa were not associated with increased rainfall or even occurred during droughts [207, 208]. Human activities such as irrigation and dam construction were suggested to have contributed to these events by providing suitable larval habitat [182].

In their study from Kenya, Hightower et al. [209] showed that while rainfall and derived measures such as NDVI are important indicators of RVF incidence in humans, there are in fact a number of geological, geographical and meteorological factors that together create the optimal conditions for RVFV occurrence. Specifically, RVF incidence was higher in (i) arid regions (< 700 mm annual rainfall) that had received more rainfall than other parts of the country prior to an RVFV outbreak; (ii) areas characterized by lower elevations (< 500 m), due to the limited abundance of vectors at higher elevations; (iii) flat landscapes with depressions that transform into water pools following heavy rainfall, thereby providing larval habitat; (iv) areas with soil textures that retain water easily, facilitating rehydration of mosquito eggs; and (v) areas with dense bush vegetation that provides mosquitoes with landing zones and resting areas [209].

Other recent studies also showed that RVFV occurrence was positively associated with vegetation cover [210, 211], the water holding capacity of soil (clay and loam were significant risk factors; [212, 213] and increased rainfall (> 405 mm during the previous 2 months; [213]). Likewise, Glancey et al. [205] argued that while rainfall and NDVI are indicative of epizootic RVFV outbreaks in South Africa, other ecological factors probably play a role as well, including close proximity to water bodies and surrounding land use, particularly shrubland, grassland, or agricultural areas. In Madagascar, recurrent transmission of RVFV was associated with a wet, temperate climate, high altitude, paddy fields and vicinity to a dense forest [214]. Thus, a range of environmental factors that provide suitable mosquito habitat at each life stage contribute to RVFV emergence [209].

As for all mosquito-borne viruses, temperature also plays an important role in the ecology of RVFV [201, 210, 211]. Higher temperatures (17–35 °C) allow mosquito populations to increase more rapidly, reaching large, stable population sizes for longer periods of time [122, 129], and facilitates viral replication and transmission in both *Aedes* spp. [215] and *Culex* spp. [216] mosquitoes.

Finally, the emergence and spread of RVFV across Africa and beyond is also linked to economic activities, in particular trade and movement of ruminant livestock and goods [25, 197, 217]. For example, phylogenetic analyses of virus isolated from Yemen and Saudi Arabia revealed that the emergence of RVFV in the Arabian Peninsula after 2000 was probably caused by the introduction of infected livestock from the African continent [206, 218, 219]. In Tanzania, Sudan, and Madagascar, animal movement into other ecological zones, where large populations of competent *Culex* mosquito species were already present, amplified outbreaks by creating secondary foci of RVFV [196, 214, 220]. Trade in goods also poses a risk, as it may aid the introduction of infected *Aedes* mosquito eggs, whereas infected adults may be transported by aircraft or cargo [217, 221]. Thus, both environmental and social factors are important to consider when assessing the risk of emergence of RVFV [206].

## Discussion

We systematically reviewed 183 studies on the ecological risk factors associated with the circulation of six priority arboviruses (JEV, WNV, RVFV, CCHFV, TBEV and LIV) that are of considerable concern to northwestern Europe (Fig. 1). Climate, habitat, animal density and movement were most often correlated with increased disease risk, with each of these factors contributing to virus circulation either directly or indirectly *via* their impacts on the virus, its vectors and/or hosts (see Additional file 2: Table S1). These factors can therefore be used for risk-based surveillance programs, although the strength and direction of their relationship with disease risk sometimes differs between viruses and may depend on local conditions.

Climatic factors were identified as important drivers for arbovirus outbreaks and sustained arbovirus circulation through a direct effect on vector capacity and, in the case of TBEV, on the occurrence of interstadial co-feeding. However, the relationships between climatic conditions and arbovirus outbreaks were not always consistent due to spatial and temporal differences in local biotic and abiotic factors [153, 222]. For example, both negative and positive relationships with precipitation were found for RVFV, JEV and CCHFV. Laboratory experiments can help elucidate the underlying mechanisms and explain correlations observed in the field. Such experiments have shown that vector competence increases with temperature in some mosquito biotypes but not in others [139], and that some virus strains are better adapted to warmer climates than others [150]. Similar optimum conditions exist for precipitation. Rising temperatures as predicted under current climate change scenarios have often been hypothesized to change current patterns of disease incidence and distribution [222, 223]. In Europe, many vectors and pathogens are expected to expand both northwards and to higher latitudes, e.g. *I. ricinus*, *H. marginatum*, WNV [35, 224, 225], while exotic arboviruses like RVFV may become established [226, 227]. However, the potential role of climate change in the distribution and incidence of some arboviruses like TBEV remains disputed [84–87] or poorly investigated like JEV [228]. It is likely that while some areas in Europe become more favorable for some of these vectors and/or pathogens, others become less favorable with rising temperatures.

Specific types of vegetation or land use were often found to be associated with increased disease risk through their impacts on suitable habitat for vectors and (reservoir) hosts, thereby increasing vector capacity. Agricultural practices such as the creation of rice paddies, abandonment of farmlands, and a high rate of habitat fragmentation may provide ideal breeding sites for vectors, increase host and vector densities and contact rates, and stimulate host and vector movement between habitat patches [17, 43].

Animal density and movement contributes to viral spread, threatening areas where competent vectors are already present or being co-introduced. Wetlands or other habitats where migratory birds concentrate in large flocks therefore pose a relatively larger risk for the introduction of CCHFV-infected ticks and/or that of bird-associated arboviruses such as WNV or JEV [56, 134, 229]. Livestock trading has been implicated in the spread of RVFV [25], CCHFV [230] and LIV [118] throughout the western Palaearctic region. Some mosquito species can maintain WNV, JEV and RVFV *via* vertical transmission (adults to eggs) [129, 231], so that trade of goods, like used car tires and ornamental plants harboring mosquito eggs and/or larvae can favor long-distance dispersal of both virus and mosquito species to novel areas [232–234]. Virus introduction and local transmission through travel of viremic people to non-endemic areas has been documented for various arboviruses as recently illustrated again for chikungunya virus in France and Italy [5, 235]. However, as humans are dead-end hosts for the arboviruses in this review, human travel is unlikely to play a role in the spread of these arboviruses.

Studies on drivers of arbovirus outbreaks typically correlate ecological variables to disease incidence rates in humans and/or animals, so that the underlying causal mechanisms remain unclear in many of these studies. For example, climate may not be the single most important factor driving the recent temporal patterns in the epidemiology of some vector-borne diseases, as human activities that increases risk of exposure (e.g. harvesting of forest products) are often strongly confounded with climatic variables [86, 89]. Therefore, the perceived relationships between climate and clinical cases may be better explained by human behavior than with vector or virus ecology [73]. Socio-economic changes that increase human activities in risky forest habitats (e.g. hunting practices, animal husbandry, collecting mushrooms) can increase exposure regardless of the abundance of infected vectors [86]. This stresses the need of directly studying the impact of environmental conditions on vectors and arboviruses, both in the field and through laboratory experiments [139, 151] while studying the influence of human behavior on risk of exposure.

Nevertheless, certain sets of ecological conditions that may facilitate the introduction and subsequent establishment of the six arboviruses discussed here can be used to target surveillance in areas where these viruses have yet to establish. These include abiotic conditions, habitat characteristics, and abundances of competent vectors and (reservoir) hosts (Additional file 2: Table S1). Specifically, local abundances of migratory birds returning from Africa and/or the Mediterranean can be used to identify areas where WNV or CCHFV-infected *Hyalomma* ticks may be introduced [56, 236], whereas data on point-to-point international trade of livestock originating from endemic areas is relevant for identifying locations of potential introduction of RVFV, LIV, and CCHFV-infected ticks [25, 237]. For arbovirus establishment, data on local densities of competent reservoir hosts (e.g. ruminant livestock for RVFV and CCHFV, ardeid birds and pigs for JEV, wetland birds and corvids for WNV, sheep for LIV) and arthropod vectors (e.g. *Culex* and *Aedes* mosquitoes, *I. ricinus* and *Hyalomma* ticks), as well as particular vegetation types (e.g. habitat fragmentation and shrubland for CCHFV, broad-leaf and mixed forests for TBEV, water bodies for JEV, WNV and RVFV) are relevant for targeting surveillance. Finally, differences in local abiotic conditions between endemic areas and areas of potential emergence, such as temperature, humidity, and precipitation, are important to consider when selecting surveillance sites.

## Conclusions

Our review reflects the complex ecology of vector-borne diseases, with the establishment of sustained transmission and the emergence of human and/or animal disease influenced by multiple factors. Zoonotic vector-borne diseases may be present well before human illness is observed in routine (syndrome-based) surveillance programs and are therefore amenable to early warning surveillance, which could lead to timely risk management. There is an urgent need for both cost-effective and efficient surveillance programmes that focus sampling in the right place at the right time, ideally targeting multiple arboviruses and vectors simultaneously. Implementing such surveillance programmes requires extensive knowledge on the ecological factors driving increased densities of infected vectors. The current review therefore provides baseline information on suitable ecological conditions under which outbreaks of six priority arboviruses can occur. The many common denominators between the arboviruses discussed here should facilitate a focused surveillance effort, targeting multiple arboviruses in specific high-risk areas. However, existing differences in the specific climatic conditions, habitat type, and host species that are important for each specific arbovirus, will determine the extent to which high-risk areas of different arboviruses overlap and hence whether the targeting of multiple arboviruses simultaneously is feasible in an adequate manner enabling sensitive early-warning.

## Additional files

**Additional file 1.** Background on each virus.

**Additional file 2: Table S1.** Ecological risk factors for each virus.

## Data Availability

All data generated or analyzed during this study are included in this published article and its additional files.

## Abbreviations

WNV: West Nile virus
JEV: Japanese encephalitis virus
RVFV: Rift Valley fever virus
TBEV: tick-borne encephalitis virus
LIV: louping-ill virus
CCHFV: Crimean-Congo hemorrhagic fever virus
PRISMA: Preferred Reporting Items for Systematic Reviews and Meta-Analyses
RH: relative humidity
NDVI: Normalized Difference Vegetation Index
SD: saturation deficit
R_0_: basic reproduction number
AMT: annual mean temperature
ENM: ecological niche modelling
DWSI: Disease Water Stress Index
ENSO: El Niño/Southern Oscillation phenomenon
