# Supplementary material for: Risk factors associated with sustained circulation of six zoonotic arboviruses: a systematic review for selection of surveillance sites in non-endemic areas
Source: Parasit Vectors. 2019 May 27;12:265. doi: 10.1186/s13071-019-3515-7 (PMC6537422; doi:10.1186/s13071-019-3515-7)
Supplement: Supplementary file 1 — Additional file 1. Background on each virus. [file 13071_2019_3515_MOESM1_ESM.docx]

# Additional file 1

## Background on each virus

CCHFV: Crimean-Congo hemorrhagic fever (genus *Orthonairovirus*, family *Nairoviridae*) is a highly contagious zoonosis with the largest geographic distribution of all tick-borne viruses, affecting people in parts of Africa, Asia, Eastern Europe, and the Middle East ([Bente et al. 2013](#_ENREF_9)). Although the virus is known to infect a wide range of both wild and domestic animals, it causes severe disease only in humans and new-born mice ([Whitehouse 2004](#_ENREF_96)). Smaller wildlife species, such as hares and hedgehogs, are suspected to act as reservoir hosts ([Appannanavar and Mishra 2011](#_ENREF_3)). Patients become infected through tick bites or direct contact with blood, bodily fluids, or tissue from infected animals while human-to-human transmission seems limited to nosocomial infection ([Bente et al. 2013](#_ENREF_9), [Papa et al. 2015](#_ENREF_71)). The combination of the extensive area over which CCHFV occurs, its high case fatality rate (ranging from 2-80%; ([Whitehouse 2004](#_ENREF_96), [Leblebicioglu 2010](#_ENREF_55)), and recurring nosocomial outbreaks make the virus an important human pathogen ([Ergönül 2006](#_ENREF_21), [Mardani and Keshtkar-Jahromi 2007](#_ENREF_61)). Moreover, both the incidence and geographic distribution of clinical cases of CCHFV have increased over the past 15 years ([Estrada-Peña and de la Fuente 2014](#_ENREF_23)), with the first two autochthonous human cases reported in Spain in 2016 ([García Rada 2016](#_ENREF_27)).

CCHFV has been isolated from over 30 species of ticks, and vector competence has been demonstrated for various species of the genera *Amblyomma, Dermacentor*, and *Rhipicephalus* ([Turell 2007](#_ENREF_89)). However, the most efficient and common vectors are considered to be *Hyalomma* spp. ticks, whose global distribution coincides with the occurrence of CCHFV ([Ergönül 2006](#_ENREF_21), [Turell 2007](#_ENREF_89)). Transmission occurs both transstadially (i.e. from larva to nymph to adult) and transovarially (i.e. from infected adult females to their eggs) across seasons, suggesting that *Hyalomma* spp. ticks act as both vector and reservoir of CCHFV ([Turell 2007](#_ENREF_89)). A recent modelling study concluded that transovarial transmission is essential for the maintenance of CCHFV circulation ([Estrada‐Peña et al. 2013](#_ENREF_24)). In addition, ticks can become infected by feeding from viraemic hosts or through co-feeding with an infected tick on a non-viraemic host ([Gordon et al. 1993](#_ENREF_31)). The principal vector, *Hyalomma* *marginatum* s.l., is a two-host tick, with larvae and nymphs feeding from the same host individual and adults feeding from different hosts and species ([Knight et al. 1978](#_ENREF_44)). Immature stages typically parasitize a large variety of birds and small- to medium-sized mammals, whereas adult ticks mainly feed from large ungulates ([Estrada-Peña and de la Fuente 2014](#_ENREF_23)).

TBEV: With 10,00-12,000 new cases each year, tick-borne encephalitis (genus *Flavivirus*, family *Flaviviridae*) is considered to be among the most significant tick-borne diseases in Europe and Eurasia ([Randolph and Rogers 2000](#_ENREF_79), [WHO 2011](#_ENREF_97)). There are three viral subtypes; Western European subtype, Siberian subtype and Far Eastern subtype. It affects the human central nervous system, causing acute and severe meningitis, encephalitis, and/or myelitis ([Ytrehus et al. 2013](#_ENREF_100)). Depending on the subtype, 10% to 30% of infected persons develop clinical symptoms, and the fatality rate for symptomatic cases is typically around 1-2% but up to 40% for the Far Eastern subtype ([Randolph and Rogers 2000](#_ENREF_79), [Mansfield et al. 2009](#_ENREF_59)). Humans may become infected after being bitten by an infected tick or, less commonly, by drinking raw milk from infected goats or sheep ([Randolph and Rogers 2000](#_ENREF_79)). TBE incidence in Europe has increased over the past two decades, with endemic areas expanding northwards, westwards and to higher latitudes ([Uzcátegui et al. 2012](#_ENREF_90)). Recently, TBEV was detected in ticks and roe deer in the Netherlands and shortly after the first two clinical cases were reported ([de Graaf et al. 2016](#_ENREF_18), [Jahfari et al. 2017](#_ENREF_40), [Weststrate et al. 2017](#_ENREF_95)).

Enzootic cycles of TBEV are maintained in rodent-tick cycles, with *Ixodes ricinus* and *I. persulcatus* as main vectors in the western and eastern Palearctic respectively ([Labuda and Randolph 1999](#_ENREF_52)). These generalist tick species feed on a wide range of vertebrates, but the principal hosts for larvae are small rodents, particularly *Apodemus* spp., *Microtus* spp., and *Myodes* spp., whereas nymphs mainly feed on small rodents and thrushes, and adult stages feeding on deer and other ungulates ([Randolph et al. 1999](#_ENREF_78), [Hofmeester et al. 2016](#_ENREF_37)). Small rodents develop short viremia of only 2-3 days and acquire immunity against TBEV after infection, so that the probability of rodent-tick transmission is quite low ([Labuda et al. 1997](#_ENREF_50)). The key to maintenance of TBEV transmission cycles is the synchronous seasonal activity of immature tick stages that allows for co-feeding between infected nymphs and uninfected larvae on non-viraemic rodent hosts ([Labuda et al. 1993](#_ENREF_51), [Labuda et al. 1996](#_ENREF_49), [Randolph et al. 1996](#_ENREF_76), [Labuda et al. 1997](#_ENREF_50), [Randolph et al. 1999](#_ENREF_78), [Randolph et al. 2000](#_ENREF_77), [Perkins et al. 2003](#_ENREF_73)). The ability of these small rodents, particularly *Apodemus* spp. to successfully feed large quantities of ticks makes them the most important amplifying hosts for tick abundances and, indirectly, TBEV transmission ([Labuda and Randolph 1999](#_ENREF_52)). In addition, TBEV infection may also be transmitted transovarially in ticks and vertically in rodent hosts, albeit at low efficiency ([Labuda and Randolph 1999](#_ENREF_52), [Uzcátegui et al. 2012](#_ENREF_90)).

LIV: Louping-ill virus (genus *Flavivirus*, family *Flaviviridae*) causes a tick-borne disease that is transmitted by the sheep tick *I. ricinus*. The virus causes encephalomyelitis in unvaccinated livestock, particularly sheep but occasionally also cattle, and is a major cause of mortality in red grouse (*Lagopus scoticus*) in endemic areas ([Ytrehus et al. 2013](#_ENREF_100), [Jeffries et al. 2014](#_ENREF_41)). Although mainly restricted to the upland areas of the British Isles, members of the LIV-group have also been reported in Spain, Norway, Turkey and Bulgaria ([Beck et al. 2013](#_ENREF_8), [Ytrehus et al. 2013](#_ENREF_100), [Jeffries et al. 2014](#_ENREF_41)). Livestock fatality rates in endemic areas range from 5% to 10%, but can reach 60% in newly introduced individuals such as weaned lambs that were moved to hill pastures ([Watts et al. 2009](#_ENREF_94), [Jeffries et al. 2014](#_ENREF_41)). Human cases of LIV almost exclusively occur in people with occupational exposure, such as veterinarians, butchers, sheep farmers, but also laboratory scientists working with LIV ([Ytrehus et al. 2013](#_ENREF_100), [Jeffries et al. 2014](#_ENREF_41)). While most human infections are asymptomatic or result in flu-like symptoms, more severe neurological effects can arise that are similar to those of TBEV. Fatal cases are rare. No cases of LIV infection in humans have been reported in Great Britain over the past 20 years, despite numerous cases of encephalitis of unknown origin ([Davison et al. 2003](#_ENREF_17)). Lack of awareness among clinicians and/or lack of specific testing may contribute to potential underreporting of human LIV cases ([Jeffries et al. 2014](#_ENREF_41)).

LIV is part of the TBEV complex and is most closely related to the European subtype ([Ytrehus et al. 2013](#_ENREF_100)). Despite their genetic similarity, the ecology of LIV transmission is rather different from TBEV. Small mammals such as rodents do not play an important role in the LIV transmission cycle ([Gilbert et al. 2000](#_ENREF_29)). The main tick hosts in the LIV system are sheep, red deer (*Cervus elaphus*), red grouse (*Lagopus scoticus*) and mountain hares (*Lepus timidus*), which differ in their susceptibility to disease, their ability to transmit infection to uninfected ticks, and their role in maintaining high tick population densities ([Jones et al. 1997](#_ENREF_43), [Watts et al. 2009](#_ENREF_94)). Only sheep and red grouse develop sufficiently high levels of viremia to support the systemic route of virus transmission ([Hudson et al. 1995](#_ENREF_39)). Mountain hares allow efficient transmission of LIV from infected to uninfected ticks via co-feeding, despite the absence of viremia ([Jones et al. 1997](#_ENREF_43)). Red deer do not produce viremia nor allow co-feeding transmission, but they are the main host for the reproductive stages of the tick and therefore greatly amplify the tick population ([Jones et al. 1997](#_ENREF_43), [Gilbert et al. 2000](#_ENREF_29)). While LIV is transmitted transstadially, no evidence of transovarial transmission has been found to date ([Jeffries et al. 2014](#_ENREF_41)).

JEV: Japanese encephalitis virus (genus *Flavivirus*, family *Flaviviridae*) is a mosquito-borne virus that is endemic throughout Southeast Asia and the Asian Pacific Rim ([Misra and Kalita 2010](#_ENREF_64)). Despite the existence of vaccines, JEV continues to be the leading cause of viral encephalitis in Asia, with over 3 billion people at risk ([Erlanger et al. 2009](#_ENREF_22), [Ghosh and Basu 2009](#_ENREF_28)). In symptomatic patients, case fatality rates can reach up to 30%, with 50% of the patients suffering permanent neurological damage ([Ghosh and Basu 2009](#_ENREF_28)). Five different genotypes are currently known, which are associated with different geographical regions and climates ([Ghosh and Basu 2009](#_ENREF_28), [Schuh et al. 2013](#_ENREF_85)). While approximately half of all JEV cases occur in China ([Campbell et al. 2011](#_ENREF_11)), the area affected by JEV has increased substantially over the past decades and the virus continues to expand its geographical range ([Johansen et al. 2001](#_ENREF_42), [Misra and Kalita 2010](#_ENREF_64)). Given the propensity of JEV and related flaviviruses (e.g., Zika virus, West Nile virus) to spread and the vector competence of mosquito species outside endemic areas, JEV could potentially be introduced and establish in the Americas, Europe, and Africa ([Van den Hurk et al. 2001](#_ENREF_91), [Ghosh and Basu 2009](#_ENREF_28), [Nett et al. 2009](#_ENREF_70), [De Wispelaere et al. 2017](#_ENREF_19)). For example, sequences identical to genomic fragments of the virus have been identified in bird tissue and mosquito samples in Italy ([Platonov et al. 2012](#_ENREF_75), [Ravanini et al. 2012](#_ENREF_80)) while recently the first autochthonous human case has been reported in Angola ([Simon-Loriere et al. 2017](#_ENREF_86)).

The current distribution of JEV coincides with that of its primary vector, *Culex tritaeniorhynchus*, although other species of the genera *Culex*, *Aedes*, and *Anopheles* have also been described as competent vectors ([Johansen et al. 2001](#_ENREF_42), [Van den Hurk et al. 2001](#_ENREF_91), [Miller et al. 2012](#_ENREF_63), [Su et al. 2014](#_ENREF_88)). The virus exists in a transmission cycle in which pigs are the main reservoir host and source of infection for humans via mosquitoes, and with birds being important hosts for the enzootic maintenance and spread of JEV ([Ghosh and Basu 2009](#_ENREF_28), [Bai et al. 2014](#_ENREF_5), [Su et al. 2014](#_ENREF_88)). Although the virus has been isolated from over 90 species of wild and domestic birds, ardeid birds (e.g., cattle egrets and pond herons) are considered to be the primary enzootic source of JEV ([Bai et al. 2014](#_ENREF_5)). In addition, JEV was isolated from bats in China, while flying foxes in Australia were shown to be able to infect recipient mosquitoes, suggesting a potential role for bats in JEV maintenance ([van den Hurk et al. 2009](#_ENREF_92), [Liu et al. 2013](#_ENREF_58)). Other domestic animals and humans are considered dead-end hosts due to low and short viremia ([Ghosh and Basu 2009](#_ENREF_28), [Misra and Kalita 2010](#_ENREF_64)). Most animal species remain asymptomatic after JEV infection, but horses may suffer from fatal encephalitis, while fetal wastage may occur in pigs. The virus can be transmitted vertically in mosquitoes, albeit inefficiently, which is a possible explanation for the overwintering capacity of the virus between epidemics ([Ghosh and Basu 2009](#_ENREF_28), [Misra and Kalita 2010](#_ENREF_64)).

WNV: West Nile Virus (genus *Flavivirus*, family *Flaviviridae*) is the most widespread arthropod-borne virus in the world, and is currently expanding its native range in Europe, Asia and Africa, as well as its introduced range in the Americas ([Kramer et al. 2008](#_ENREF_48), [Bakonyi et al. 2013](#_ENREF_6), [David and Abraham 2016](#_ENREF_16)). Although the majority (~80%) of infections in humans remain asymptomatic, about 20% of patients develop febrile illness and approximately 1% suffer from a severe, potentially fatal, neuro-invasive disease ([Artsob et al. 2009](#_ENREF_4), [Bakonyi et al. 2013](#_ENREF_6)). WNV is also of considerable veterinary concern, with about 10% of symptomatic horses developing acute encephalitis or meningitis ([Beck et al. 2013](#_ENREF_8)).

Patterns of emergence differ markedly between continents. In North America, tens of thousands of humans and horses have been infected since the introduction of WNV in 1999 ([Beck et al. 2013](#_ENREF_8)). WNV also inflicts considerable morbidity and mortality in birds and other wildlife in the USA ([Bowen and Nemeth 2007](#_ENREF_10)), even leading to dramatic declines in some bird species (e.g., American crows, *Corvus brachyrhynchos* ([LaDeau et al. 2007](#_ENREF_53)). In contrast, mass mortality among birds has not been recorded in Europe, even though carrion crows, jackdaws, and house sparrows have experimentally been shown to be highly susceptible to WNV infection ([Del Amo et al. 2014](#_ENREF_20), [Lim et al. 2014](#_ENREF_57), [Lim et al. 2015](#_ENREF_56)). Moreover, European outbreaks tend to be restricted to southeastern and eastern countries, despite seemingly favorable environmental conditions and widespread circulation of the virus in wildlife and sentinel animals in other parts of Europe ([Kramer et al. 2008](#_ENREF_48), [Bakonyi et al. 2013](#_ENREF_6), [Sambri et al. 2013](#_ENREF_84), [Marcantonio et al. 2015](#_ENREF_60)). Variation in WNV strain virulence, host susceptibility, herd immunity, vector competence, and/or past exposure to other Flaviviruses have been suggested to explain these continental differences, but the definite role of these drivers in the epidemiological pattern of WNV in Europe needs further elucidation ([Fros et al. 2015](#_ENREF_26), [Koraka et al. 2016](#_ENREF_47)). Low temperature has been suggested as the key limiting factor to further northwards spread of WNV in Europe ([Vogels et al. 2017](#_ENREF_93)).

Natural transmission of WNV occurs in a bird-mosquito-bird cycle, with virus spill-over to humans and horses as dead-end hosts ([Chaskopoulou et al. 2016](#_ENREF_12)). The virus has been detected in at least 326 species of birds and over 30 species of non-avian hosts in the United States alone ([Petersen et al. 2013](#_ENREF_74)). Vector competence has been demonstrated in 65 species of mosquitoes, but the principal bridge vectors transmitting virus between birds and humans are *Culex spp.* mosquitoes, which may also function as overwintering reservoir host ([Petersen et al. 2013](#_ENREF_74), [David and Abraham 2016](#_ENREF_16)). Transovarial transmission of WNV by female *Culex spp* has also been found ([Goddard et al. 2003](#_ENREF_30)). In Europe, the most important WNV vectors are mosquitoes belonging to the *Cx. pipiens* complex, and, to a lesser degree, *Cx. modestus* and *Cx. perixiguus* ([Hubálek and Halouzka 1999](#_ENREF_38), [Muñoz et al. 2012](#_ENREF_68), [Mughini-Gras et al. 2014](#_ENREF_66)). Both habitat and host preferences vary between mosquito vectors, with major implications for transmission risk to humans ([Marcantonio et al. 2015](#_ENREF_60)).

WNV has also been identified in six genera of ticks ([Higgs et al. 2004](#_ENREF_34)) and one biting midge species ([Naugle et al. 2004](#_ENREF_69)). However, experimentally infected *I. pacificus*, *I. ricinus*, *I. scapularis*, *Amblyomma americanum*, *D. andersoni*, and *D. variabilis* ticks were shown to be unable to maintain or transmit the virus to uninfected hosts ([Anderson et al. 2003](#_ENREF_1), [Lawrie et al. 2004](#_ENREF_54), [Reisen et al. 2007](#_ENREF_81)). So far, laboratory vector competence has only been demonstrated for the tick *H. marginatum* ([Formosinho and Santos-Silva 2005](#_ENREF_25)), but as their WNV prevalence in the field appears to be very low ([Mumcuoglu et al. 2005](#_ENREF_67), [Hagman et al. 2014](#_ENREF_32), [Kolodziejek et al. 2014](#_ENREF_45)), they are unlikely to play a major role in WNV epidemiology. On the other hand, *Ornithodoros moubata* ticks have been shown to be able to maintain WNV infection for 132 days in a laboratory setting and to non-systemically transmit the virus to uninfected ticks via co-feeding, so that the potential role of ticks as reservoir hosts merits further investigation ([Lawrie et al. 2004](#_ENREF_54)).

Although mammals are susceptible to WNV infection, most species (including humans and horses) are considered dead-end hosts ([Bowen and Nemeth 2007](#_ENREF_10)). However, some mammals (rodents, rabbits) and reptiles (alligators) have been shown to develop sufficient viremia to infect feeding mosquitoes, questioning their status as dead-end hosts in the WNV transmission cycle ([Higgs et al. 2005](#_ENREF_33), [Root et al. 2006](#_ENREF_83), [Bowen and Nemeth 2007](#_ENREF_10), [McGee et al. 2007](#_ENREF_62), [Kramer et al. 2008](#_ENREF_48)). Further, there is strong evidence for non-vector transmission among birds via the fecal-oral route and through predation or consumption of infected carrion ([Komar et al. 2003](#_ENREF_46), [Bowen and Nemeth 2007](#_ENREF_10)). In humans, non-vector transmission can occur via blood transfusion, breast feeding, and organ transplantation ([Kramer et al. 2008](#_ENREF_48)). Clearly, the transmission ecology of WNV is complex and particularly poorly understood in the Old World. Simultaneous circulation of multiple WNV lineages with varying pathogenicity renders quantification of the ecological parameters involved in the transmission cycle even more challenging ([Marcantonio et al. 2015](#_ENREF_60)).

RVFV: Rift Valley Fever Virus (genus *Phlebovirus*, family *Phenuiviridae*) is a mosquito-borne virus pathogenic to a wide range of wild mammals, domestic livestock, and humans. Periodic epidemics and epizootics occur in most sub-Saharan African countries, with recent outbreaks in the Arabian Peninsula ([Balkhy and Memish 2003](#_ENREF_7), [Himeidan et al. 2014](#_ENREF_36), [Sindato et al. 2014](#_ENREF_87)). Although less fatal for humans than other hemorrhagic fevers such as Ebola and Marburg, RVFV outbreaks can destroy local economies due to mass abortion and mortality in livestock, particularly in younger animals, and imposed restrictions on animal movement and trade ([Anyamba et al. 2001](#_ENREF_2), [Balkhy and Memish 2003](#_ENREF_7)).

Several other features make RVFV an important zoonosis with a high potential to spread to Europe and other parts of Asia ([Balkhy and Memish 2003](#_ENREF_7), [Clements et al. 2007](#_ENREF_15), [Moutailler et al. 2008](#_ENREF_65), [Chevalier 2013](#_ENREF_13), [Rolin et al. 2013](#_ENREF_82)). First, the virus can be transmitted by a wide range of vectors that have global distributions, including mosquitoes, ticks and flies (Pepin et al. 2010, and references therein). The most important of these vectors are *Aedes* spp. and *Culex* spp. mosquitoes, which function as reservoir/maintenance vectors and epidemic/amplifying vectors respectively ([Pepin et al. 2010](#_ENREF_72)). Second, RVFV can spread via blood or other bodily secretions from infected humans and animals ([Hightower et al. 2012](#_ENREF_35)). This places humans at risk when e.g., nursing sick people, drinking raw milk, treating infected animals, or assisting animals during abortions or birth ([Wilson et al. 1994](#_ENREF_99), [Chevalier et al. 2010](#_ENREF_14)). Third, infected livestock such as sheep and cattle become highly viraemic, facilitating viral transmission ([Balkhy and Memish 2003](#_ENREF_7)). Finally, the virus is transovarially transmitted, and drought-resistant mosquito species (e.g., *Ae. mcintoshi*, *Ae. circumluteolus*) produce infected eggs that are able to survive for years until favorable environmental conditions allow them to hatch ([Wilson 1994](#_ENREF_98), [Pepin et al. 2010](#_ENREF_72)).

## References

Anderson, J. F., A. J. Main, T. G. Andreadis, S. K. Wikel, and C. R. Vossbrinck. 2003. Transstadial transfer of West Nile virus by three species of ixodid ticks (Acari: Ixodidae). Journal of Medical Entomology **40**:528-533.

Anyamba, A., K. J. Linthicum, and C. J. Tucker. 2001. Climate-disease connections: Rift Valley fever in Kenya. Cadernos de saude publica **17**:S133-S140.

Appannanavar, S. B. and B. Mishra. 2011. An update on Crimean Congo hemorrhagic fever. Journal of global infectious diseases **3**:285.

Artsob, H., D. Gubler, D. Enria, M. Morales, M. Pupo, M. Bunning, and J. Dudley. 2009. West Nile Virus in the New World: trends in the spread and proliferation of West Nile Virus in the Western Hemisphere. Zoonoses and Public Health **56**:357-369.

Bai, Y., Z. Xu, J. Zhang, D. Mao, C. Luo, Y. He, G. Liang, B. Lu, M. S. Bisesi, and Q. Sun. 2014. Regional impact of climate on Japanese encephalitis in areas located near the three gorges dam. PLoS ONE **9**:e84326.

Bakonyi, T., E. Ferenczi, K. Erdélyi, O. Kutasi, T. Csörgő, B. Seidel, H. Weissenböck, K. Brugger, E. Bán, and N. Nowotny. 2013. Explosive spread of a neuroinvasive lineage 2 West Nile virus in Central Europe, 2008/2009. Veterinary microbiology **165**:61-70.

Balkhy, H. H. and Z. A. Memish. 2003. Rift Valley fever: an uninvited zoonosis in the Arabian peninsula. International journal of antimicrobial agents **21**:153-157.

Beck, C., M. A. Jimenez-Clavero, A. Leblond, B. Durand, N. Nowotny, I. Leparc-Goffart, S. Zientara, E. Jourdain, and S. Lecollinet. 2013. Flaviviruses in Europe: complex circulation patterns and their consequences for the diagnosis and control of West Nile disease. International Journal of Environmental Research and Public Health **10**:6049-6083.

Bente, D. A., N. L. Forrester, D. M. Watts, A. J. McAuley, C. A. Whitehouse, and M. Bray. 2013. Crimean-Congo hemorrhagic fever: History, epidemiology, pathogenesis, clinical syndrome and genetic diversity. Antiviral Research **100**:159-189.

Bowen, R. A. and N. M. Nemeth. 2007. Experimental infections with West Nile virus. Current opinion in infectious diseases **20**:293-297.

Campbell, G. L., S. L. Hills, M. Fischer, J. A. Jacobson, C. H. Hoke, J. M. Hombach, A. A. Marfin, T. Solomon, T. F. Tsai, and V. D. Tsu. 2011. Estimated global incidence of Japanese encephalitis: a systematic review. Bulletin of the World Health Organization **89**:766-774.

Chaskopoulou, A., G. L’Ambert, D. Petric, R. Bellini, M. Zgomba, T. A. Groen, L. Marrama, and D. J. Bicout. 2016. Ecology of West Nile virus across four European countries: review of weather profiles, vector population dynamics and vector control response. Parasites & Vectors **9**:482.

Chevalier, V. 2013. Relevance of Rift Valley fever to public health in the European Union. Clinical Microbiology and Infection **19**:705-708.

Chevalier, V., M. Pepin, L. Plee, and R. Lancelot. 2010. Rift Valley fever--a threat for Europe? Euro surveillance: bulletin Europeen sur les maladies transmissibles= European communicable disease bulletin **15**:19506-19506.

Clements, A. C., D. U. Pfeiffer, V. Martin, C. Pittiglio, N. Best, and Y. Thiongane. 2007. Spatial risk assessment of Rift Valley fever in Senegal. Vector-Borne and Zoonotic Diseases **7**:203-216.

David, S. and A. M. Abraham. 2016. Epidemiological and clinical aspects on West Nile virus, a globally emerging pathogen. Infectious Diseases:1-16.

Davison, K. L., N. S. Crowcroft, M. E. Ramsay, D. Brown, and N. J. Andrews. 2003. Viral encephalitis in England, 1989-1998: what did we miss? Emerging Infectious Diseases **9**:234-240.

de Graaf, J., J. Reimerink, G. Voorn, E. bij de Vaate, A. de Vries, B. Rockx, A. Schuitemaker, and V. Hira. 2016. First human case of tick-borne encephalitis infection acquired in the Netherlands, July 2016. Euro Surveill **21**:3.

De Wispelaere, M., P. Desprès, and V. Choumet. 2017. European *Aedes albopictus* and *Culex pipiens* are competent vectors for Japanese encephalitis virus. PLoS neglected tropical diseases **11**:e0005294.

Del Amo, J., F. Llorente, J. Figuerola, R. C. Soriguer, A. M. Moreno, P. Cordioli, H. Weissenböck, and M. Á. Jiménez-Clavero. 2014. Experimental infection of house sparrows (Passer domesticus) with West Nile virus isolates of Euro-Mediterranean and North American origins. Veterinary Research **45**:33.

Ergönül, Ö. 2006. Crimean-Congo haemorrhagic fever. The Lancet Infectious Diseases **6**:203-214.

Erlanger, T. E., S. Weiss, J. Keiser, J. Utzinger, and K. Wiedenmayer. 2009. Past, present, and future of Japanese encephalitis. Emerg Infect Dis **15**:1-7.

Estrada-Peña, A. and J. de la Fuente. 2014. The ecology of ticks and epidemiology of tick-borne viral diseases. Antiviral Research **108**:104-128.

Estrada‐Peña, A., F. Ruiz‐Fons, P. Acevedo, C. Gortazar, and J. la Fuente. 2013. Factors driving the circulation and possible expansion of Crimean–Congo haemorrhagic fever virus in the western Palearctic. Journal of applied microbiology **114**:278-286.

Formosinho, P. and M. Santos-Silva. 2005. Experimental infection of Hyalomma marginatum ticks with West Nile virus. Acta virologica **50**:175-180.

Fros, J. J., C. Geertsema, C. B. Vogels, P. P. Roosjen, A.-B. Failloux, J. M. Vlak, C. J. Koenraadt, W. Takken, and G. P. Pijlman. 2015. West Nile virus: high transmission rate in north-western European mosquitoes indicates its epidemic potential and warrants increased surveillance. PLoS Negl Trop Dis **9**:e0003956.

García Rada, A. 2016. First outbreak of Crimean-Congo haemorrhagic fever in western Europe kills one man in Spain. BMJ **354**.

Ghosh, D. and A. Basu. 2009. Japanese encephalitis—a pathological and clinical perspective. PLoS Negl Trop Dis **3**:e437.

Gilbert, L., L. Jones, P. Hudson, E. Gould, and H. Reid. 2000. Role of small mammals in the persistence of Louping‐ill virus: field survey and tick co‐feeding studies. Medical and Veterinary Entomology **14**:277-282.

Goddard, L. B., A. E. Roth, W. K. Reisen, and T. W. Scott. 2003. Vertical transmission of west nile virus by three california Culex (Diptera: Culicidae) species. Journal of Medical Entomology **40**:743-746.

Gordon, S. W., K. J. Linthicum, and J. Moulton. 1993. Transmission of Crimean-Congo hemorrhagic fever virus in two species of Hyalomma ticks from infected adults to cofeeding immature forms. DTIC Document.

Hagman, K., C. Barboutis, C. Ehrenborg, T. Fransson, T. G. Jaenson, P.-E. Lindgren, Å. Lundkvist, F. Nyström, J. Waldenström, and E. Salaneck. 2014. On the potential roles of ticks and migrating birds in the ecology of West Nile virus. Infection ecology & epidemiology **4**.

Higgs, S., B. S. Schneider, D. L. Vanlandingham, K. A. Klingler, and E. A. Gould. 2005. Nonviremic transmission of West Nile virus. Proceedings of the National Academy of Sciences of the United States of America **102**:8871-8874.

Higgs, S., K. Snow, and E. A. Gould. 2004. The potential for West Nile virus to establish outside of its natural range: a consideration of potential mosquito vectors in the United Kingdom. Transactions of the Royal Society of Tropical Medicine and Hygiene **98**:82-87.

Hightower, A., C. Kinkade, P. M. Nguku, A. Anyangu, D. Mutonga, J. Omolo, M. K. Njenga, D. R. Feikin, D. Schnabel, and M. Ombok. 2012. Relationship of climate, geography, and geology to the incidence of Rift Valley fever in Kenya during the 2006–2007 outbreak. The American journal of tropical medicine and hygiene **86**:373-380.

Himeidan, Y. E., E. J. Kweka, M. M. Mahgoub, E. A. El Rayah, and J. O. Ouma. 2014. Recent outbreaks of rift valley fever in east africa and the middle east. Frontiers in public health **2**.

Hofmeester, T. R., E. C. Coipan, S. E. van Wieren, H. H. T. Prins, W. Takken, and H. Sprong. 2016. Few vertebrate species dominate the Borrelia burgdorferi s.l. life cycle. Environmental Research Letters **11**:043001.

Hubálek, Z. and J. Halouzka. 1999. West Nile fever--a reemerging mosquito-borne viral disease in Europe. Emerging Infectious Diseases **5**:643.

Hudson, P. J., R. Norman, M. K. Laurenson, D. Newborn, M. Gaunt, L. Jones, H. Reid, E. Gould, R. Bowers, and A. Dobson. 1995. Persistence and transmission of tick-borne viruses: Ixodes ricinus and louping-ill virus in red grouse populations. Parasitology **111**:S49-S58.

Jahfari, S., A. de Vries, J. M. Rijks, S. Van Gucht, H. Vennema, H. Sprong, and B. Rockx. 2017. Tick-borne encephalitis virus in ticks and roe deer, the Netherlands. Emerging Infectious Diseases **23**:1028.

Jeffries, C., K. Mansfield, L. Phipps, P. Wakeley, R. Mearns, A. Schock, S. Bell, A. Breed, A. Fooks, and N. Johnson. 2014. Louping ill virus: an endemic tick-borne disease of Great Britain. Journal of General Virology **95**:1005-1014.

Johansen, C., A. Pyke, P. Zborowski, D. Phillips, J. Mackenzie, and S. Ritchie. 2001. Entomological investigations of an outbreak of Japanese encephalitis virus in the Torres Strait, Australia, in 1998. Journal of Medical Entomology **38**:581-588.

Jones, L. D., M. Gaunt, R. S. Hails, K. Laurenson, P. J. Hudson, H. Reid, P. Henbest, and E. A. Gould. 1997. Transmission of louping ill virus between infected and uninfected ticks co-feeding on mountain hares. Medical and Veterinary Entomology **11**:172-176.

Knight, M., R. Norval, and Y. Rechav. 1978. The life cycle of the tick Hyalomma marginatum rufipes Koch (Acarina: Ixodidae) under laboratory conditions. The Journal of Parasitology:143-146.

Kolodziejek, J., M. Marinov, B. J. Kiss, V. Alexe, and N. Nowotny. 2014. The complete sequence of a West Nile virus lineage 2 strain detected in a Hyalomma marginatum marginatum tick collected from a song thrush (Turdus philomelos) in eastern Romania in 2013 revealed closest genetic relationship to strain Volgograd 2007. PLoS ONE **9**:e109905.

Komar, N., S. Langevin, S. Hinten, N. Nemeth, E. Edwards, D. Hettler, B. Davis, R. Bowen, and M. Bunning. 2003. Experimental infection of North American birds with the New York 1999 strain of West Nile virus. Emerging Infectious Diseases **9**:311-322.

Koraka, P., L. Barzon, and B. Martina. 2016. West Nile Virus Infections in (European) Birds. J Neuroinfect Dis **7**:2.

Kramer, L. D., L. M. Styer, and G. D. Ebel. 2008. A global perspective on the epidemiology of West Nile virus. Annu. Rev. Entomol. **53**:61-81.

Labuda, M., J. M. Austyn, E. V. A. Zuffova, O. T. O. Kozuch, N. Fuchsberger, J. A. N. Lysy, and P. A. Nuttall. 1996. Importance of Localized Skin Infection in Tick-Borne Encephalitis Virus Transmission. Virology **219**:357-366.

Labuda, M., O. Kozuch, E. Zuffová, E. Elecková, R. S. Hails, and P. A. Nuttall. 1997. Tick-borne encephalitis virus transmission between ticks cofeeding on specific immune natural rodent hosts. Virology **235**:138-143.

Labuda, M., P. Nuttall, O. Kožuch, E. Elečková, T. Williams, E. Žuffová, and A. Sabo. 1993. Non-viraemic transmission of tick-borne encephalitis virus: a mechanism for arbovirus survival in nature. Experientia **49**:802-805.

Labuda, M. and S. E. Randolph. 1999. Survival strategy of tick-borne encephalitis virus: Cellular basis and environmental determinants. Zentralblatt für Bakteriologie **289**:513-524.

LaDeau, S. L., A. M. Kilpatrick, and P. P. Marra. 2007. West Nile virus emergence and large-scale declines of North American bird populations. Nature **447**:710-713.

Lawrie, C. H., N. Y. Uzcátegui, E. A. Gould, and P. A. Nuttall. 2004. Ixodid and argasid tick species and West Nile virus. Emerg Infect Dis **10**:653-657.

Leblebicioglu, H. 2010. Crimean–Congo haemorrhagic fever in Eurasia. International journal of antimicrobial agents **36**:S43-S46.

Lim, S. M., A. C. Brault, G. van Amerongen, A. M. Bosco-Lauth, H. Romo, V. D. Sewbalaksing, R. A. Bowen, A. D. Osterhaus, P. Koraka, and B. E. Martina. 2015. Susceptibility of carrion crows to experimental infection with lineage 1 and 2 West Nile viruses. Emerging Infectious Diseases **21**:1357.

Lim, S. M., A. C. Brault, G. van Amerongen, V. D. Sewbalaksing, A. D. Osterhaus, B. E. Martina, and P. Koraka. 2014. Susceptibility of European jackdaws (*Corvus monedula*) to experimental infection with lineage 1 and 2 West Nile viruses. Journal of General Virology **95**:1320-1329.

Liu, S., X. Li, Z. Chen, Y. Chen, Q. Zhang, Y. Liao, J. Zhou, X. Ke, L. Ma, and J. Xiao. 2013. Comparison of genomic and amino acid sequences of eight Japanese encephalitis virus isolates from bats. Archives of virology **158**:2543-2552.

Mansfield, K., N. Johnson, L. Phipps, J. Stephenson, A. Fooks, and T. Solomon. 2009. Tick-borne encephalitis virus–a review of an emerging zoonosis. Journal of General Virology **90**:1781-1794.

Marcantonio, M., A. Rizzoli, M. Metz, R. Rosà, G. Marini, E. Chadwick, and M. Neteler. 2015. Identifying the environmental conditions favouring West Nile Virus outbreaks in Europe. PLoS ONE **10**:e0121158.

Mardani, M. and M. Keshtkar-Jahromi. 2007. Crimean-Congo hemorrhagic fever. Archives of Iranian medicine **10**:204-214.

McGee, C. E., B. S. Schneider, Y. A. Girard, D. L. Vanlandingham, and S. Higgs. 2007. Nonviremic transmission of West Nile Virus: Evaluation of the effects of space, time, and mosquito species. The American journal of tropical medicine and hygiene **76**:424-430.

Miller, R. H., P. Masuoka, T. A. Klein, H.-C. Kim, T. Somer, and J. Grieco. 2012. Ecological niche modeling to estimate the distribution of Japanese encephalitis virus in Asia. PLoS Negl Trop Dis **6**:e1678.

Misra, U. K. and J. Kalita. 2010. Overview: japanese encephalitis. Progress in neurobiology **91**:108-120.

Moutailler, S., G. Krida, F. Schaffner, M. Vazeille, and A.-B. Failloux. 2008. Potential Vectors of Rift Valley Fever Virus in the Mediterranean Region. Vector-Borne and Zoonotic Diseases **8**:749-754.

Mughini-Gras, L., P. Mulatti, F. Severini, D. Boccolini, R. Romi, G. Bongiorno, C. Khoury, R. Bianchi, F. Montarsi, and T. Patregnani. 2014. Ecological niche modelling of potential West Nile virus vector mosquito species and their geographical association with equine epizootics in Italy. EcoHealth **11**:120-132.

Mumcuoglu, K. Y., C. Banet-Noach, M. Malkinson, U. Shalom, and R. Galun. 2005. Argasid ticks as possible vectors of West Nile virus in Israel. Vector-Borne & Zoonotic Diseases **5**:65-71.

Muñoz, J., S. Ruiz, R. Soriguer, M. Alcaide, D. S. Viana, D. Roiz, A. Vázquez, and J. Figuerola. 2012. Feeding patterns of potential West Nile virus vectors in south-west Spain. PLoS ONE **7**:e39549.

Naugle, D. E., C. L. Aldridge, B. L. Walker, T. E. Cornish, B. J. Moynahan, M. J. Holloran, K. Brown, G. D. Johnson, E. T. Schmidtmann, and R. T. Mayer. 2004. West Nile virus: pending crisis for greater sage‐grouse. Ecology Letters **7**:704-713.

Nett, R., G. Campbell, and W. Reisen. 2009. Potential for the emergence of Japanese encephalitis virus in California. Vector-Borne and Zoonotic Diseases **9**:511-517.

Papa, A., A. Mirazimi, I. Köksal, A. Estrada-Pena, and H. Feldmann. 2015. Recent advances in research on Crimean-Congo hemorrhagic fever. Journal of Clinical Virology **64**:137-143.

Pepin, M., M. Bouloy, B. H. Bird, A. Kemp, and J. Paweska. 2010. Rift Valley fever virus (Bunyaviridae: Phlebovirus): an update on pathogenesis, molecular epidemiology, vectors, diagnostics and prevention. Veterinary Research **41**:61.

Perkins, S. E., I. M. Cattadori, V. Tagliapietra, A. P. Rizzoli, and P. J. Hudson. 2003. Empirical evidence for key hosts in persistence of a tick-borne disease. International Journal for Parasitology **33**:909-917.

Petersen, L. R., A. C. Brault, and R. S. Nasci. 2013. West Nile virus: review of the literature. Jama **310**:308-315.

Platonov, A., G. Rossi, L. Karan, K. Mironov, L. Busani, and G. Rezza. 2012. Does the Japanese encephalitis virus (JEV) represent a threat for human health in Europe? Detection of JEV RNA sequences in birds collected in Italy. Euro Surveill **17**:32.

Randolph, S., L. Gern, and P. Nuttall. 1996. Co-feeding ticks: epidemiological significance for tick-borne pathogen transmission. Parasitology Today **12**:472-479.

Randolph, S., R. Green, M. Peacey, and D. Rogers. 2000. Seasonal synchrony: the key to tick-borne encephalitis foci identified by satellite data. Parasitology **121**:15-23.

Randolph, S., D. Miklisova, J. Lysy, D. Rogers, and M. Labuda. 1999. Incidence from coincidence: patterns of tick infestations on rodents facilitate transmission of tick-borne encephalitis virus. Parasitology **118**:177-186.

Randolph, S. E. and D. J. Rogers. 2000. Fragile transmission cycles of tick-borne encephalitis virus may be disrupted by predicted climate change. Proceedings of the Royal Society of London B: Biological Sciences **267**:1741-1744.

Ravanini, P., E. Huhtamo, V. Ilaria, M. Crobu, A. Nicosia, L. Servino, F. Rivasi, S. Allegrini, U. Miglio, and A. Magri. 2012. Japanese encephalitis virus RNA detected in Culex pipiens mosquitoes in Italy. Euro Surveill **17**:20221.

Reisen, W., A. Brault, V. Martinez, Y. Fang, K. Simmons, S. Garcia, E. Omi-Olsen, and R. Lane. 2007. Ability of transstadially infected Ixodes pacificus (Acari: Ixodidae) to transmit West Nile virus to song sparrows or western fence lizards. Journal of Medical Entomology **44**:320-327.

Rolin, A. I., L. Berrang-Ford, and M. A. Kulkarni. 2013. The risk of Rift Valley fever virus introduction and establishment in the United States and European Union. Emerging microbes & infections **2**:e81.

Root, J. J., P. T. Oesterle, N. M. Nemeth, K. Klenk, D. H. Gould, R. G. Mclean, L. Clark, and J. S. Hall. 2006. Experimental infection of fox squirrels (Sciurus niger) with West Nile virus. The American journal of tropical medicine and hygiene **75**:697-701.

Sambri, V., M. Capobianchi, R. Charrel, M. Fyodorova, P. Gaibani, E. Gould, M. Niedrig, A. Papa, A. Pierro, G. Rossini, S. Varani, C. Vocale, and M. P. Landini. 2013. West Nile virus in Europe: emergence, epidemiology, diagnosis, treatment, and prevention. Clinical Microbiology and Infection **19**:699-704.

Schuh, A. J., M. J. Ward, A. J. L. Brown, and A. D. Barrett. 2013. Phylogeography of Japanese encephalitis virus: genotype is associated with climate. PLoS Negl Trop Dis **7**:e2411.

Simon-Loriere, E., O. Faye, M. Prot, I. Casademont, G. Fall, M. D. Fernandez-Garcia, M. M. Diagne, J.-M. Kipela, I. S. Fall, E. C. Holmes, A. Sakuntabhai, and A. A. Sall. 2017. Autochthonous Japanese Encephalitis with Yellow Fever Coinfection in Africa. New England Journal of Medicine **376**:1483-1485.

Sindato, C., E. D. Karimuribo, D. U. Pfeiffer, L. E. Mboera, F. Kivaria, G. Dautu, B. Bernard, and J. T. Paweska. 2014. Spatial and temporal pattern of Rift Valley fever outbreaks in Tanzania; 1930 to 2007. PLoS ONE **9**:e88897.

Su, C.-L., C.-F. Yang, H.-J. Teng, L.-C. Lu, C. Lin, K.-H. Tsai, Y.-Y. Chen, L.-Y. Chen, S.-F. Chang, and P.-Y. Shu. 2014. Molecular epidemiology of Japanese encephalitis virus in mosquitoes in Taiwan during 2005–2012. PLoS Negl Trop Dis **8**:e3122.

Turell, M. 2007. Role of Ticks in the Transmission of Crimean-Congo Hemorrhagic Fever Virus. Pages 143-154 *in* O. Ergonul and C. Whitehouse, editors. Crimean-Congo Hemorrhagic Fever. Springer Netherlands.

Uzcátegui, N. Y., T. Sironen, I. Golovljova, A. E. Jääskeläinen, H. Välimaa, Å. Lundkvist, A. Plyusnin, A. Vaheri, and O. Vapalahti. 2012. Rate of evolution and molecular epidemiology of tick-borne encephalitis virus in Europe, including two isolations from the same focus 44 years apart. Journal of General Virology **93**:786-796.

Van den Hurk, A., D. Nisbet, C. Johansen, P. Foley, S. Ritchie, and J. Mackenzie. 2001. Japanese encephalitis on Badu Island, Australia: the first isolation of Japanese encephalitis virus from Culex gelidus in the Australasian region and the role of mosquito host-feeding patterns in virus transmission cycles. Transactions of the Royal Society of Tropical Medicine and Hygiene **95**:595-600.

van den Hurk, A. F., C. S. Smith, H. E. Field, I. L. Smith, J. A. Northill, C. T. Taylor, C. C. Jansen, G. A. Smith, and J. S. Mackenzie. 2009. Transmission of Japanese Encephalitis virus from the black flying fox, Pteropus alecto, to Culex annulirostris mosquitoes, despite the absence of detectable viremia. The American journal of tropical medicine and hygiene **81**:457-462.

Vogels, C. B. F., G. P. Göertz, G. P. Pijlman, and C. J. M. Koenraadt. 2017. Vector competence of northern and southern European Culex pipiens pipiens mosquitoes for West Nile virus across a gradient of temperatures. Medical and Veterinary Entomology:n/a-n/a.

Watts, E., S. Palmer, A. Bowman, R. Irvine, A. Smith, and J. Travis. 2009. The effect of host movement on viral transmission dynamics in a vector-borne disease system. Parasitology **136**:1221-1234.

Weststrate, A. C., D. Knapen, G. D. Laverman, B. Schot, J. J. Prick, S. A. Spit, J. Reimerink, B. Rockx, and F. Geeraedts. 2017. Increasing evidence of tick-borne encephalitis (TBE) virus transmission, the Netherlands, June 2016. Eurosurveillance **22**.

Whitehouse, C. A. 2004. Crimean–Congo hemorrhagic fever. Antiviral Research **64**:145-160.

WHO. 2011. Vaccines against tick-borne encephalitis: WHO position paper–Recommendations. Vaccine **29**:8769-8770.

Wilson, M. L. 1994. Rift Valley fever virus ecology and the epidemiology of disease emergencea. Annals of the New York Academy of Sciences **740**:169-180.

Wilson, M. L., L. E. Chapman, D. B. Hall, E. A. Dykstra, K. Ba, H. G. Zeller, M. Traore-Lamizana, J.-P. Hervy, K. J. Linthicum, and C. Peters. 1994. Rift Valley fever in rural northern Senegal: human risk factors and potential vectors. American Journal of Tropical Medicine and Hygiene **50**:663-675.

Ytrehus, B., K. Vainio, S. G. Dudman, J. Gilray, and K. Willoughby. 2013. Tick-borne encephalitis virus and louping-ill virus may co-circulate in Southern Norway. Vector-Borne and Zoonotic Diseases **13**:762-768.
